# Non-use and inadequate use of cervical cancer screening among a representative sample of women in the United States

**DOI:** 10.3389/fpubh.2024.1321253

**Published:** 2024-04-22

**Authors:** Marie-Rachelle Narcisse, Pearl A. McElfish, Emily Hallgren, Natalie Pierre-Joseph, Holly C. Felix

**Affiliations:** ^1^Department of Psychiatry and Human Behavior, Brown University, Providence, RI, United States; ^2^College of Medicine, University of Arkansas for Medical Sciences Northwest, Springdale, AR, United States; ^3^Department of Pediatrics, Boston University Chobanian and Avedisian School of Medicine, Boston, MA, United States; ^4^Department of Pediatrics, Boston Medical Center, Boston, MA, United States; ^5^Fay W. Boozman College of Public Health, University of Arkansas for Medical Sciences, Little Rock, AR, United States

**Keywords:** cervical cancer screening, Andersen's Behavioral Model of Health Services Use, National Health Interview Survey, United States, predisposing, enabling, needs factors

## Abstract

**Introduction:**

Women's adherence to the United States (U.S.) Preventive Services Task Force guidelines for cervical cancer screening was determined by examining predisposing, enabling, and needs factors from Andersen's Behavioral Model of Health Services Use conceptual framework.

**Methods:**

The outcome was operationalized as cervical cancer screening use, non-use, and inadequate-use. Multinomial logistic regression was conducted on data from the 2019 National Health Interview Survey of 7,331 eligible women aged 21–65.

**Results:**

Compared with women who used cervical cancer screening services, women aged 30–65 were less likely to be Non-Users than those aged 21–29. Hispanic, Asian, and American Indian/Alaska Native (AIAN) women were more likely to be Non-Users than White women. More educated women were less likely to be Non-Users. Foreign-born women <10 years in the U.S. were more likely to be Non-Users than U.S.-born women. Women with financial hardship were less likely to be Non-Users. Poorer women and uninsured women were more likely to be Non-Users. Women with children in their household were less likely to be Non-Users than those without children. Women who had a well-visit in the past year were less likely to be Non-Users. Women with a history of human papillomavirus (HPV) vaccination were less likely to be Non-Users. Compared with women who used cervical cancer screening services, women aged 30–65 were less likely to be Inadequate-Users. AIAN women were more likely to be Inadequate-Users. Women of other races were less likely to be Inadequate-Users. Employed women were less likely to be Inadequate-Users. Uninsured women were more likely to be Inadequate-Users. Women who had a well-visit within a year were less likely to be Inadequate-Users. Women with past HPV vaccination were more likely to be Inadequate-Users. Smokers were less likely to be Inadequate-Users.

**Discussion:**

Predisposing, enabling, and needs factors are differently associated with non-use and inadequate use of cervical cancer screening. Understanding factors associated with the use, non-use, and inadequate use of cervical cancer screening is crucial to avoid or curb unnecessary tests, increased costs to both society and individuals, and the ill-allocation of limited resources.

## 1 Introduction

In 2020, nearly 300,000 women had cervical cancer in the United States (U.S.). In 2023, about 13,963 women were newly diagnosed with cervical cancer, and 4,310 died of cervical cancer (1).

The vast majority of cervical cancer (>95%) is caused by the human papillomavirus (HPV), the most common sexually transmitted infection in the U.S. (2). One in 10 people in the U.S. develops persistent HPV infections, putting them at risk for cervical cancer. HPV vaccination can prevent 90% of cervical cancers from occurring. With an average total expenditure per patient of $56,250 during the first year after diagnosis—reaching $97,000 annually at the end-of-life—cervical cancer treatment costs are considerable for society (3, 4). Thus, early detection is crucial for reducing cervical cancer deaths and alleviating the economic burden associated with treatment (5).

Women who are not appropriately screened are at the highest risk of developing cervical cancer (6–8). Cervical cancer can be detected with three types of screenings: (1) an HPV test; (2) a Papanicolaou test (also called a Pap smear or cervical cytology); and (3) an HPV/Pap co-test that combines HPV and Pap tests. Effective strategies ensuring all women are screened at appropriate intervals are essential for reducing U.S. cervical cancer incidence and mortality. To that end, the U.S. Preventive Services Task Force (USPSTF) has developed specific guidelines for cervical cancer screening based on women's risk and age: every 3 years with a Pap test for women ages 21–29; every 3 years with a Pap test for women ages 30–65; every 5 years for those with high-risk HPV testing, or every 5 years with both tests. The USPSTF recommends against cervical cancer screening for women aged <21, those with a history of hysterectomy, those without a history of cervical cancer or high-grade precancerous lesion, those aged >65, those who have had adequate screening, and women with low risk for cervical cancer. See Table 1 for the most recent 2018 guidelines (9, 10).

**Table 1:** USPSTF guidelines for cervical cancer screening.

**Population**	**Recommendation**	**Grade**
Women aged 21–65 years	The USPSTF recommends screening for cervical cancer every 3 years with cervical cytology alone in women aged 21 to 29 years. For women aged 30 to 65 years, the USPSTF recommends screening every 3 years with cervical cytology alone, every 5 years with hrHPV testing alone, or every 5 years with hrHPV testing in combination with cytology (cotesting). See the Clinical Considerations section for the relative benefits and harms of alternative screening strategies for women 21 years or older	A
Women younger than 21 years	The USPSTF recommends against screening for cervical cancer in women younger than 21 years	D
Women who have had a hysterectomy	The USPSTF recommends against screening for cervical cancer in women who have had a hysterectomy with removal of the cervix and do not have a history of a high-grade precancerous lesion (i.e., cervical intraepithelial neoplasia [CIN] grade 2 or 3) or cervical cancer	D
Women older than 65 years	The USPSTF recommends against screening for cervical cancer in women older than 65 years who have had adequate prior screening and are not otherwise at high risk for cervical cancer. See the Clinical Considerations section for discussion of adequate prior screening and risk factors that support screening after age 65 years	D

Source: https://www.uspreventiveservicestaskforce.org/uspstf/document/ClinicalSummaryFinal/cervical-cancer-screening.

hrHPV, high-risk human papillomavirus; USPSTF, The United States Preventive Services Task Force.

These recommendations apply to individuals who have a cervix, regardless of their sexual history or HPV vaccination status. These recommendations do not apply to individuals who have been diagnosed with a high-grade precancerous cervical lesion or cervical cancer, those with in utero exposure to diethylstilbestrol, or those who have a compromised immune system (e.g., individuals living with HIV).

For a summary of the evidence systematically reviewed in making this recommendation, the full recommendation statement, and supporting documents, please go to https://www.uspreventiveservicestaskforce.org.

Although these guidelines can improve cervical cancer detection and prevention, screening services can be costly, with costs varying widely due to screening under-use and inadequate use (3, 11–15). A previous study has identified an excess cost of $166,100 over 5 years for each ineligible woman (e.g., <21 and >65 years or those with a hysterectomy) who underwent screening (15). Equally important is identifying unscreened women among the *eligible* population to ensure equitable access to treatments and reduce excess costs. Therefore, research that identifies factors associated with use, inadequate use, and non-use of cervical cancer screenings is urgent for public health and economic imperatives, as well as achieving equitable access to screenings for hard-to-reach and underserved women.

Screening rates are below the *Healthy People 2030* targets of 79.2% (73.9% in 2021) based on the most recent guidelines (*objective C-09*) and 11.5% (from 5.3% in 2020) for receipt of appropriate evidence-based clinical preventive services (*objective AHS-08*) (10). Multiple sociodemographic, healthcare access, and health status factors such as race/ethnicity, education and income levels, marital status, sexual orientation, and rurality (16–18) contribute to use, inadequate use, and non-use of cervical cancer screening rates in certain populations. While previous studies have primarily examined factors predicting cervical cancer screening in silos, fewer studies have employed a robust theoretical framework to investigate the confluence of various sociodemographic, healthcare access, and health factors in explaining adherence to USPSTF cervical cancer screening guidelines among women in the U.S. Our study fills this gap by examining predisposing, enabling, and needs factors contributing the most to disparities in cervical cancer screening rates, and more specifically, the use, non-use, and inadequate use of cervical cancer screenings.

### 1.1 Theoretical framework, research objectives and hypotheses

To better understand non-adherence to the USPSTF cervical cancer guidelines, we applied Andersen's Behavioral Model of Health Services Use (Andersen's Model) (19, 20) by examining predisposing, enabling, and needs factors associated with use, non-use, or inadequate use of cervical cancer screening services guidelines among age-eligible women in the U.S. More specifically, predisposing factors refer to socio-demographic characteristics that “predispose” women to adhere cervical cancer screening guidelines. Enabling factors are those that “enable” or, to the contrary, impede women's adherence to these guidelines. Needs factors are subjective or objective health needs that incite women to get screened. We hypothesized a significant association between these factors and non-use or inadequate use of cervical cancer screening services among women eligible for cervical cancer screening.

## 2 Materials and methods

### 2.1 Data source

This study is based on a secondary data analysis of the National Health Interview Survey (NHIS, 2019) of noninstitutionalized and civilian participants living in 50 states and the District of Columbia. NHIS is an annual survey that compiles cross-sectional data on physical and mental health status, functioning, health insurance coverage, health services utilization, and sociodemographic characteristics of the U.S. population. For this study, the Adult file was used. This file contains data obtained from an adult aged 18 or older randomly selected from a household. A suitable proxy is chosen if the selected participant is incapable of responding due to physical or mental limitations (21).

### 2.2 Study population and inclusion criteria

**Figure 1:**
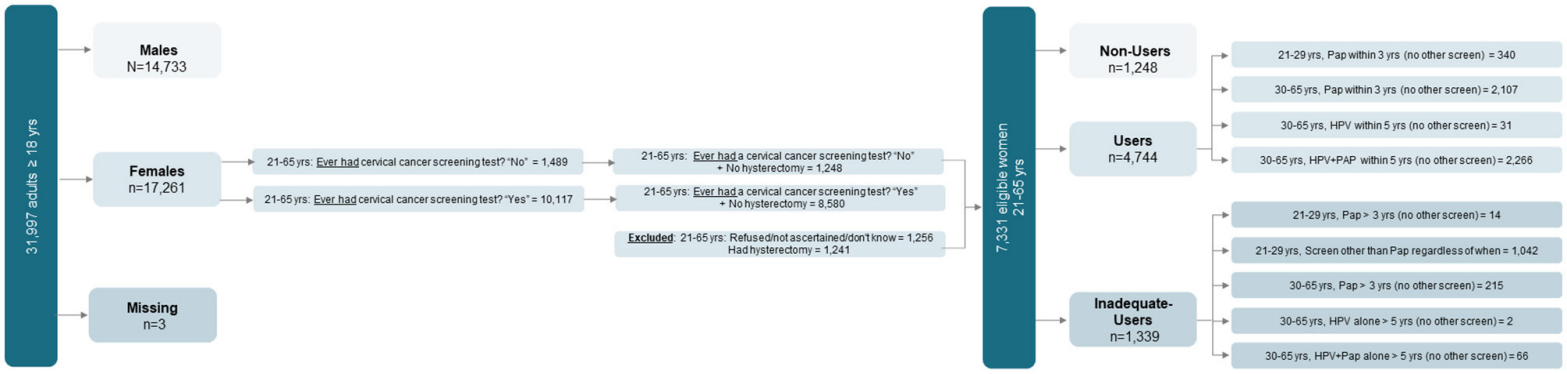
Flow chart of eligible women classified as Users, Non-Users, and Inadequate-Users based on adherence to the United States Preventive Services Task Force (USPSTF) guidelines for cervical cancer screening. Source: 2019 National Health Interview Survey (NHIS)—National Center of Health Statistics, Centers for Diseases Control and Prevention.

According to the 2019 NHIS, 31,997 adults aged ≥18 (17,261 women) were interviewed. Among them, 10,117 women aged 21–65 reported having had a cervical cancer screening test, and 1,489 reported not having had the test. Among them, 8,580 age-eligible women without a hysterectomy had a cervical cancer screening, whereas 1,248 age-eligible women without a hysterectomy did not. After consideration of inclusion/exclusion criteria and missing data, the final analytical sample included 7,331 women aged 21–65, including (1) a first group (the *Users*) composed of 4,744 age-eligible women who were properly screened (recommended screening method) within the timeframe recommended by USPSTF; (2) a second group (the *Non-Users*) composed of 1,248 age-eligible women without a hysterectomy who could have been screened but were not; and (3) a third group (the *Inadequate-Users)* composed of 1,339 age-eligible women who were non-adherent either because they were improperly screened or untimely screened (i.e., outside of the time window recommend by USPSTF). See Figure 1 for a flow chart of the study sample.

### 2.3 Measures

#### 2.3.1 Outcome

The dependent variable is a three-category variable that captures: (1) all age-eligible women who were adherent *Users* (i.e., between the ages of 21–29 who had a Pap test within 3 years with no other screening; or between the ages of 30–65 who had a Pap test within 3 years with no other screening; or between the ages of 30–65 who had HPV within 5 years with no other screening; or 30–65 years who had Pap + HPV co-screening within 5 years with no other screening); (2) all age-eligible women who were *Non-Users*; and (3) all eligible women who were *Inadequate-Users*.

#### 2.3.2 Explanatory variables

Predisposing (age 21–29 vs. 30–65, sex, sexual orientation, race/ethnicity, education, marital status, region), enabling (employment status, federal poverty level [FPL], public assistance, health insurance coverage, medical financial hardship, usual source of care, number of children in household), and needs (wellness visits within past year, number of health care visits in the past year, self-reported health status, anxiety, past cancer diagnosis, breast examination by health professionals, mammography, HPV vaccine, disability, body mass index [BMI], smoking status).

### 2.4 Descriptive and regression analyses

To assess adherence to the USPSTF cervical cancer screening guidelines among eligible participants, percentages and 95% confidence intervals (CI) for categorical variables and means and standard errors (SE) for continuous variables were computed.

After running a multicollinearity test to detect among explanatory variables, mammography, usual sources of care, and breast examination by health professionals were excluded based on the variance inflation factor (VIF). The test was recomputed among the remaining variables and generated a mean VIF of 1.46. Multinomial logistic regression—with odds ratios (OR) and 95% CIs—was used to estimate the odds of being a *Non-user* or an *Inadequate-User* vs. a *User* accounting for the women's predisposing, enabling, and needs factors. The group of eligible women who followed USPSTF guidelines (the adherent *Users*) was used as the base outcome. All analyses employed sampling weights, stratification, and primary sampling unit variables to account for NHIS complex design by using the *svy* function in STATA/SE 17 (22).

## 3 Results

Six in 10 eligible women (59.8%) were adherent *Users* (met the USPSTF screening guidelines), two in 10 were *Non-Users* (19.5%), and two in 10 were *Inadequate-Users* (20.7%). The mean age of adherent *Users* was 44.8 years; *Non-Users* and *Inadequate-Users* were younger (average age of 40.4 and 31.3 years, respectively). Other predisposing, enabling, and needs characteristics of adherent *Users, Non-Users, and Inadequate-Users* are presented in Table 2.

**Table 2:** Descriptive analysis: cervical cancer screening among eligible women aged 21–65. Predisposing, enabling, and needs factors among Users, Non-Users, and Inadequate-Users based on the USPSTF cervical cancer guidelines: NHIS, 2019.

	**Percentages (95% CI)** ^**†**^		
	**Users** ***N*** = **4,744**	**Non-Users** ***N*** = **1,248**	**Inadequate-Users** ***N*** = **1,339**
	59.8 (58.4–61.3)	19.5 (18.2–20.8)	20.7 (19.5–21.9)
**Predisposing factors**			
**Age**			
Age (mean and 95% CI)	44.8 (43.3–45.3)	40.4 (39.9–41.5)	31.3 (30.4–32.2)
Age 21–29	18.0 (15.9–20.4)	25.3 (22.7–28.0)	56.6 (53.9–59.4)
Age 30–65	78.0 (76.5–79.4)	17.0 (15.6–18.4)	5.1 (4.4–5.8)
**Sexual orientation**			
Gay/lesbian	45.1 (39.0–51.4)	22.7 (17.3–29.3)	32.1 (26.3–38.5)
Straight	61.5 (60.0–63.1)	18.4 (17.1–19.8)	20.0 (18.8–21.3)
**Marital status (married/in partnership)**			
No	49.2 (46.9–51.6)	26.2 (24.1–28.5)	24.5 (22.5–26.7)
Yes	66.5 (64.8–68.2)	15.1 (13.8–16.6)	18.4 (17.0–19.8)
**Race/ethnicity**			
White	64.1 (62.2–65.9)	13.7 (12.4–15.2)	22.2 (20.7–23.8)
Black	60.4 (56.5–64.2)	20.1 (17.2–23.4)	19.5 (16.6–22.8)
Asian	55.8 (50.4–61.0)	33.8 (28.8–39.3)	10.4 (7.6–14.1)
AIAN	34.2 (22.4–48.3)	32.9 (13.8–60.0)	33.0 (15.8–56.3)
Other	59.5 (49.2–69.1)	19.1 (12.3–28.4)	21.4 (14.5–30.5)
Hispanic	50.4 (47.2–53.7)	29.2 (26.0–32.6)	20.4 (17.6–23.5)
**Education level**			
< HS/GED–HS/GED	47.2 (41.5–53.0)	36.3 (31.6–42.4)	16.0 (12.2–20.5)
Some college	51.5 (48.4–54.7)	26.0 (23.3–28.9)	22.5 (20.1–25.1)
Associate/bachelor's	55.3 (51.7–58.9)	20.1 (17.3–23.4)	24.5 (21.7–27.6)
>Bachelor's	67.8 (66.1–69.5)	12.9 (11.6–14.2)	19.3 (17.9–20.9)
**Nativity**			
Born in the U.S.	60.5 (58.8–62.3)	15.8 (14.5–17.2)	15.8 (14.5–17.2)
Not born in the U.S. but lived in the U.S. for ≤ 10 years	38.5 (32.5–44.9)	45.1 (38.7–51.6)	16.4 (12.0–22.0)
Not born in the U.S. but lived in the U.S. for >10 years	65.0 (61.8–68.1)	26.6 (23.7–29.7)	8.4 (6.7–10.5)
**Region**			
Northeast	63.9 (60.8–66.9)	19.7 (16.6–23.1)	16.4 (13.9–19.3)
Midwest	61.5 (58.5–64.5)	17.5 (15.4–19.9)	20.9 (18.4–23.8)
South	56.7 (54.2–59.2)	20.0 (17.8–22.4)	23.2 (21.4–25.2)
West	60.2 (57.1–63.2)	20.1 (17.5–23.0)	19.7 (17.4–22.2)
**Metropolitan areas**			
Large central metro	59.8 (57.4–62.1)	21.4 (19.3–23.6)	18.8 (16.9–20.9)
Large fringe metro	63.6 (60.8–66.4)	17.8 (15.4–20.4)	18.6 (16.4–21.0)
Medium and small metro	57.0 (54.3–59.7)	19.5 (17.3–22.0)	23.4 (21.4–25.6)
Non-metropolitan	58.8 (54.0–63.5)	17.5 (13.8–22.0)	23.6 (23.6–27.6)
**Enabling factors**			
**Employment status**			
Not employed last week or the past year OR never worked	61.3 (58.1–64.5)	24.5 (21.7–27.6)	14.1 (11.8–16.9)
Employed last week or the past year	59.5 (57.9–61.0)	18.4 (17.0–19.8)	22.2 (20.8–23.5)
**Problems paying or were unable to pay any medical bills**			
No	60.2 (58.6–61.8)	20.0 (18.6–21.5)	19.8 (18.5–21.1)
Yes	58.1 (54.3–61.8)	16.4 (13.8–19.4)	25.5 (22.4–25.5)
**How worried are you that you will be able to pay your medical bills**			
Not at all worried	60.8 (58.7–62.7)	18.7 (17.0–20.6)	20.5 (18.9–22.3)
Somewhat worried	61.5 (59.0–63.9)	16.9 (15.2–18.9)	21.6 (19.6–23.7)
Very worried	55.0 (51.7–58.2)	25.4 (22.5–28.7)	19.6 (17.0–22.4)
**Federal poverty level**			
≤ 138%	44.3 (41.2–47.4)	30.8 (27.7–34.0)	24.9 (22.3–27.7)
>138–250	52.0 (48.8–55.1)	24.1 (21.2–27.2)	23.9 (21.1–27.0)
>250–400	60.7 (57.6–63.8)	18.4 (15.9–21.2)	20.9 (18.5–23.5)
>400^†^	71.5 (69.5–73.4)	11.8 (10.4–13.4)	16.7 (15.0–18.6)
**Income from public assistance**			
No	60.6 (59.1–62.1)	18.9 (17.6–20.3)	20.5 (19.3–21.7)
Yes	52.2 (44.6–59.7)	22.2 (16.5–28.6)	25.8 (19.7–33.1)
**Health insurance coverage**			
Uninsured	39.2 (35.1–43.5)	35.4 (31.3–39.7)	25.4 (21.8–29.4)
Medicaid	48.9 (45.0–52.9)	25.8 (22.3–29.5)	25.3 (21.9–29.1)
Military	70.2 (60.9–78.2)	9.0 (4.2–18.3)	20.7 (14.6–28.6)
Medicare	65.9 (57.7–73.1)	25.6 (18.9–33.8)	8.5 (5.1–13.9)
Other	52.9 (47.2–58.5)	23.7 (18.6–29.7)	23.4 (18.5–29.3)
Private	65.8 (64.1–67.4)	15.1 (13.7–16.5)	19.1 (17.8–20.6)
**Usual source of medical care**			
No	36.7 (32.6–41.0)	35.9 (31.4–40.6)	27.4 (23.5–31.6)
Yes	62.2 (60.7–63.7)	17.8 (16.5–19.1)	20.0 (18.0–21.3)
**Usual source of care at what place**			
Emergency room/urgent care	64.1 (62.5–65.7)	17.0 (15.7–18.4)	18.9 (17.7–20.2)
A doctor's office or health center	51.5 (46.4–56.6)	20.0 (16.1–24.6)	28.5 (24.0–33.5)
Other places	41.0 (33.9–48.5)	31.3 (23.7–40.0)	27.7 (20.4–36.4)
**Number of children in the household**			
0	57.4 (55.5–59.2)	21.4 (19.7–23.2)	21.3 (19.7–23.0)
1	62.7 (60.1–65.3)	17.2 (15.3–19.3)	20.0 (18.1–22.1)
2+	62.2 (57.8–66.4)	18.1 (14.7–22.1)	19.7 (16.4–23.5)
**Needs factors**			
**Well-visits in the past year**			
No	35.2 (27.9–43.3)	36.9 (29.1–45.3)	27.9 (20.9–36.3)
Yes	64.4 (62.7–66.1)	16.4 (15.0–17.8)	19.2 (17.9–20.6)
**Number of times visited hospital emergency room, past 12 months**			
0	61.3 (59.7–62.9)	19.3 (17.9–20.8)	19.4 (18.0–20.8)
1	57.2 (52.8–61.5)	17.4 (14.5–20.9)	25.3 (21.9–29.1)
2+	50.7 (45.9–55.4)	24.1 (20.3–28.5)	25.2 (21.2–29.6)
**Had HPV vaccine**			
No	67.0 (65.2–68.7)	20.0 (19.2–22.2)	12.4 (11.3–13.6)
Yes	39.9 (37.1–42.7)	14.9 (12.7–17.3)	45.2 (42.3–48.2)
**Self-reported health status**			
Excellent	60.5 (58.0–63.0)	17.9 (15.8–20.1)	21.6 (19.3–24.0)
Very good	60.8 (58.5–63.0)	17.7 (15.8–19.9)	21.5 (19.5–23.6)
Good	57.5 (54.7–60.3)	22.3 (19.9–24.9)	20.1 (18.0–22.4)
Fair/poor	60.0 (55.6–64.4)	23.0 (19.2–27.3)	16.9 (13.7–20.7)
**Anxiety**			
No	61.0 (59.4–62.5)	20.1 (18.8–21.6)	18.9 (17.7–20.2)
Yes	54.5 (51.0–62.5)	16.3 (14.0–19.0)	29.2 (26.1–32.5)
**Disabled**			
No	59.9 (58.4–61.4)	19.1 (17.8–20.5)	21.0 (19.8–22.2)
Yes	57.7 (51.9–63.2)	27.0 (21.9–32.8)	15.3 (11.5–20.1)
**BMI status**			
Underweight	1.7 (1.3–2.1)	2.3 (1.6–3.4)	2.1 (1.4–3.2)
Normal weight	38.6 (36.9–40.3)	35.4 (32.1–38.9)	40.3 (37.1–43.5)
Overweight	28.2 (26.7–29.7)	29.9 (26.7–33.4)	27.5 (24.7–30.6)
Obese	31.6 (29.9–33.3)	32.3 (28.8–36.0)	30.1 (27.2–33.1)
**History of cancer**			
No	94.5 (93.6–95.3)	97.8 (96.7–98.5)	96.9 (95.7–97.7)
Yes	5.5 (4.7–6.4)	2.2 (1.5–3.3)	3.1 (2.3–4.3)
**Cigarette use**			
Smokers	11.3 (10.3–12.4)	13.4 (11.3–15.7)	12.9 (12.0–13.9)
Former-smokers	17.4 (16.2–18.7)	9.0 (7.3–11.1)	14.6 (13.7–15.6)
Never-smokers	71.3 (69.8–72.8)	77.6 (74.7–80.2)	72.5 (71.1–73.8)

Source: 2019 National Health Interview Survey (NHIS)—National Center of Health Statistics.

^†^Weighted percentages with 95% confidence intervals (CI).

BMI, body mass index; CI, confidence interval; HPV, human papillomavirus; HS/GED, high school/General Educational Development; NHIS, National Health Interview Survey; U.S., United States; USPSTF, The United States Preventive Services Task Force.

### 3.1 Explaining non-use and inadequate use of cervical cancer screening services among age-eligible women

Multinomial logistic regression was conducted to examine among eligible women (i.e., 21–65 years without a hysterectomy) the odds of being a *Non-User* vs. *User* and the odds of being an *Inadequate-User* vs. *User*, accounting for women's predisposing, enabling, and needs factors. Only the statistically significant associations pre-determined at α = 0.05 are reported below. See Table 3 for all significant and non-significant associations.

**Table 3:** Predisposing, enabling, and needs factors associated with cervical cancer screening among Users, Non-Users, and Inadequate-Users: results from multinomial logistic regression.

	**Users (base outcome)**	**Non-Users**		**Inadequate-Users**	
		**OR (95% CI)**	***p***	**OR (95% CI)**	***p***
**Predisposing factors**					
**Age**					
Age 21–29	Reference				
Age 30–65		**0.12 (0.09–0.17)**	**< 0.001**	**0.02 (0.01–0.02)**	**< 0.001**
**Sexual orientation**					
Gay/lesbian	Reference				
Straight		0.74 (0.45–1.24)	0.254	1.43 (0.72–2.82)	0.303
**Marital status (married/in partnership)**					
No	Reference				
Yes		0.79 (0.60–1.02)	0.075	1.29 (0.98–1.70)	0.073
**Race/ethnicity**					
White	Reference				
Black		1.23 (0.84–1.81)	0.282	0.74 (0.45–1.21)	0.235
Asian		**2.21 (1.31–3.72)**	**< 0.001**	0.75 (0.40–1.42)	0.370
AIAN		**8.81 (2.88–26.92)**	**< 0.001**	**4.01 (1.21–13.30)**	**0.023**
Other		1.16 (0.54–2.47)	0.703	**0.39 (0.16–0.96)**	**0.040**
Hispanic		**1.50 (1.00–2.23)**	**0.049**	0.92 (0.59–1.46)	0.734
**Education level**					
< HS/GED-HS/GED	Reference				
Some college		0.89 (0.55–1.44)	0.637	1.04 (0.54–2.03)	0.900
Associate/bachelor's		0.63 (0.37–1.07)	0.086	0.63 (0.30–1.30)	0.208
>Bachelor's		**0.47 (0.28–0.78)**	**0.004**	0.83 (0.42–1.64)	0.593
**Nativity**					
Born in the U.S.	Reference				
Not born in the U.S. but lived in the U.S. for ≤ 10 years		**1.96 (1.02–3.77)**	**0.045**	1.03 (0.47–2.28)	0.936
Not born in the U.S. but lived in the U.S. for >10 years		**1.64 (1.12–2.41)**	**0.012**	0.99 (0.61–1.61)	0.965
**Region**					
Northeast	Reference				
Midwest		0.95 (0.63–1.43)	0.808	0.86 (0.51–1.45)	0.574
South		0.97 (0.69–1.38)	0.883	1.41 (0.92–2.17)	0.118
West		1.01 (0.70–1.47)	0.938	1.16 (0.72–1.89)	0.537
**Metropolitan areas**					
Large central metro	Reference				
Large fringe metro		0.97 (0.70–1.34)	0.845	0.98 (0.66–1.46)	0.925
Medium and small metro		0.89 (0.66–1.21)	0.456	0.97 (0.70–1.35)	0.847
Non-metropolitan		0.74 (0.49–1.13)	0.163	1.23 (0.72–2.11)	0.448
**Enabling factors**					
**Employment status**					
Not employed last week or the past year OR never worked	Reference				
Employed last week or the past year		1.07 (0.77–1.49)	0.684	**0.58 (0.37–0.93)**	**0.022**
**Problems paying or were unable to pay any medical bills**					
No	Reference				
Yes		**0.60 (0.40–0.91)**	**0.016**	1.19 (0.77–1.84)	0.421
**How worried are you that you will be able to pay your medical bills**					
Not at all worried	Reference				
Somewhat worried		0.86 (0.65–1.13)	0.271	1.23 (0.92–1.65)	0.168
Very worried		1.02 (0.71–1.46)	0.916	0.89 (0.55–1.46)	0.646
**Federal poverty level**					
≤ 138%		**1.74 (1.07–2.82)**	**0.025**	1.38 (0.80–2.35)	0.243
>138–250		**1.52 (1.02–2.28)**	**0.042**	1.23 (0.77–1.95)	0.381
>250–400		**1.38 (1.00–1.91)**	**0.048**	1.22 (0.83–1.79)	0.304
>400	Reference				
**Income from public assistance**					
No	Reference				
Yes		0.67 (0.35–1.29)	0.231	1.03 (0.53–2.00)	0.926
**Health insurance coverage**					
Uninsured		**1.91 (1.20–3.02)**	**0.006**	**2.09 (1.15–3.79)**	**0.016**
Medicaid		1.33 (0.87–2.04)	0.183	1.16 (0.70–1.94)	0.558
Military		0.53 (0.16–1.75)	0.298	0.72 (0.33–1.55)	0.393
Medicare		1.21 (0.49–3.00)	0.673	0.23 (0.03–1.92)	0.170
Other	Reference				
Private		1.00 (0.60–1.65)	0.997	1.31 (0.70–2.45)	0.403
**Usual source of care at what place**					
Emergency room/urgent care	Reference				
A doctor's office or health center		0.96 (0.59–1.56)	0.872	0.78 (0.44–1.39)	0.399
Other places		1.11 (0.60–2.03)	0.740	0.95 (0.45–2.00)	0.855
**Number of children in the household**					
0	Reference				
1		**0.66 (0.49–0.88)**	**0.005**	0.83 (0.60–1.14)	0.241
2+		**0.59 (0.36–0.97)**	**0.038**	1.12 (0.66–1.88)	0.679
**Needs factors**					
**Well-visits in the past year**					
No	Reference				
Yes		**0.40 (0.21–0.75)**	**0.004**	**0.24 (0.10–0.54)**	**0.001**
**Number of times visited hospital emergency room, past 12 months**					
0	Reference				
1		1.09 (0.76–1.56)	0.637	1.07 (0.72–1.60)	0.728
2+		1.25 (0.78–2.00)	0.348	0.88 (0.51–1.49)	0.627
**Had HPV vaccine**					
No	Reference				
Yes		**0.61 (0.43–0.87)**	**0.007**	**1.73 (1.29–2.32)**	**< 0.001**
**Self-reported health status**					
Excellent	Reference				
Very good		1.03 (0.76–1.38)	0.866	0.94 (0.68–1.29)	0.684
Good		1.17 (0.81–1.69)	0.394	0.95 (0.66–1.36)	0.779
Fair/poor		1.02 (0.62–1.69)	0.925	0.79 (0.41–1.56)	0.502
**Anxiety**					
No	Reference				
Yes		1.26 (0.92–1.72)	0.151	1.27 (0.88–1.83)	0.194
**Disabled**					
No	Reference				
Yes		1.31 (0.75–2.29)	0.347	1.31 (0.61–2.83)	0.488
**BMI status**					
Underweight		1.04 (0.44–2.43)	0.933	0.95 (0.40–2.27)	0.910
Normal weight	Reference				
Overweight		1.15 (0.85–1.56)	0.358	1.18 (0.84–1.66)	0.328
Obese		1.01 (0.74–1.36)	0.961	1.04 (0.72–1.50)	0.823
**History of cancer**					
No	Reference				
Yes		0.68 (0.39–1.20)	0.184	1.14 (0.58–2.24)	0.695
**Cigarette use**					
Smokers	Reference				
Former-smokers		0.92 (0.35–1.29)	0.696	0.88 (0.51–1.51)	0.630
Never-smokers		0.92 (0.35–1.29)	0.679	**0.50 (0.32–0.80)**	**0.004**

Source: 2019 National Health Interview Survey (NHIS)—National Center of Health Statistics.

Usual source of care was removed from regression analysis because of multicollinearity with places of usual source of care. Statistically significant associations at α = 0.05 are bolded.

BMI, body mass index; CI, confidence interval; HPV, human papillomavirus; HS/GED, high school/General Educational Development; NHIS, National Health Interview Survey; OR, odds ratio; U.S., United States; USPSTF, The United States Preventive Services Task Force.

#### 3.1.1 The Non-Users vs. Users

##### 3.1.1.1 Predisposing factors

Compared with women aged 21–29, those aged 30–65 had lesser odds of being *Non-Users* than *Users* (OR: 0.12, CI: 0.09–0.17). Being of Hispanic, Asian, and AIAN race/ethnicity vs. White increased the odds of being a *Non-User* (OR: 1.50, CI: 1.00–2.23; OR: 2.21, CI: 1.31–3.72; OR: 8.81, CI: 2.88–26.92, respectively). Having more than a bachelor's degree vs. <high school/General Educational Development decreased the odds of being a *Non-User* (OR: 0.47, CI: 0.28–0.78). Foreign-born women with <10 years in the U.S. had twice the odds of being *Non-Users* than those born in the U.S. (OR: 1.96, CI: 1.02–3.77), and these odds were also higher for those with ≥10 years in the U.S. (OR: 1.64, CI: 1.12–2.41).

##### 3.1.1.2 Enabling factors

Women who reported having problems paying medical bills had lesser odds of being *Non-Users* as compared with those without such hardships (OR: 0.60, CI: 0.40–0.91). Compared with women at 400% FPL, those at ≤138%, >138%−250%, and >250%−400% FPL had increased odds of being *Non-Users* (OR: 1.74, CI: 1.07–2.82); (OR: 1.52, CI: 1.02– 2.28); (OR: 1.38, CI: 1.00–1.91). Uninsured women vs. those with private insurance had greater odds of being *Non-Users* (OR: 1.91, CI: 1.20–3.02). Compared with women without children in the household, those with 1 or 2+ children had lesser odds of being *Non-Users* [(OR: 0.66, CI: 0.49–0.88) and (OR: 0.59, CI: 0.36–0.97), respectively].

##### 3.1.1.3 Needs factors

Past-year well visits were associated with decreased odds of being *Non-Users* (OR: 0.40, CI: 0.21–0.75). Past HPV vaccination was also associated with lesser odds of being *Non-Users* (OR: 0.61, CI: 0.43–0.87).

#### 3.1.2 The Inadequate-Users vs. Users

##### 3.1.2.1 Predisposing factors

Women aged 30–65 had lesser odds of being *Inadequate-Users* of cervical cancer services than their younger counterparts (OR: 0.02, CI: 0.01–0.02). Whereas AIAN women had increased odds of being *Inadequate-Users* (OR: 4.01, CI: 1.21–12.30), those of “other” races had decreased odds of being *Inadequate-Users* than White women (OR: 0.39, CI: 0.16–0.96).

##### 3.1.2.2 Enabling factors

Employed women had greater odds of being *Inadequate-Users* than unemployed ones (OR: 0.58, CI: 0.37–0.93). Uninsured women had twice the odds of being *Inadequate-Users* than their counterparts with private insurance coverage (OR: 2.09, CI: 1.15–3.79).

##### 3.1.2.3 Needs factors

Past-year well visits were associated with decreased odds of being *Inadequate-Users* (OR: 0.24, CI: 0.10–0.54). Past HPV vaccination was associated with increased odds of being *Inadequate-Users* (OR: 1.73, CI: 1.29–2.32). Never-smokers had more decreased odds of being *Inadequate-Users* than smokers (OR: 0.50, CI: 0.32–0.80).

## 4 Discussion

Cervical cancer kills thousands of women every year. Screening is a double-edged sword; over-screening limits screening services availability for eligible women (especially underserved women) (23). Under-screening puts eligible women at higher risk for undiagnosed cervical cancer and related mortality. The goal of this study was to determine factors associated with population-level non-use and inadequate use of cervical cancer screenings among eligible women using a robust theoretical framework.

Our findings showed age as a significant predisposing factor of non-use and inadequate use among eligible women. Women aged 30–65 years were less likely to be *Non-Users* or *Inadequate-Users* than their younger counterparts. Research on age has largely focused on whether screening outside the eligible age window (<21 or >65) should be expanded (24–26). A previous study based on 2013 and 2015 NHIS data showed the proportion of women aged 41–70 not recently screened increased with age, especially among women approaching the “stopping” age (27). Our finding that 21–29 year old women were more likely to adhere to the USPSTF guidelines suggests a trend reversal, as a 2000–2010 report from the Centers for Disease Control and Prevention (CDC) showed the proportion of women who never had a Pap test increased from 6.6% to 9.0% among those aged 22–30 who should have been screened every 3 years (18). However, experts have underlined the importance for continued screening among younger populations—especially the 21–25 year-old group—at higher risk for underscreening due to many barriers (e.g., fragmented healthcare, uninsurance or under-insurance, low employment, and diminutions of pelvic examinations for cervical cancer screening despite recommendations) (28).

Our study also found racial/ethnic differences in non-use and inadequate use among minority groups. AIAN women were more likely to be *Non-Users and Inadequate-Users*, whereas Asian and Hispanic women were more likely to be *Non-Users* relative to White women. Previous studies have revealed women receiving screening tests outside of guidelines were more likely to identify as Black and Hispanic (16, 23). However, our study showed Black women were not more or less likely to be *Non-Users* or *Inadequate-Users* than their White counterparts. The stark White-Black differences in death by cervical cancer may be partly explained by the fact that care continuity after screening is often inadequate for Black women vs. White women though the former group is screened for cervical cancer at rates similar to the latter groups. Differences in treatment may also be important contributing factors. Black women are 41% more likely to have cervical cancer than White women and are 75% more likely to die from it (10, 29). Among all racial/ethnic groups, black women have the lowest 5-year relative survival rate of cervical adenocarcinoma despite being the group with lowest incidence rates (30). This indicates the urgent need to examine factors other than uptake of screening programs (e.g., differential access to treatments, mechanistic influence of enabling and needs factors among this population) that may be at the core of these observed inequities in cervical cancer outcomes.

Our finding that foreign-born women with <10 years of residence in the U.S. were also more likely to be *Non-Users* is consistent with a previous research study based on pooled NHIS data (2005, 2008, 2013, 2015) finding that foreign-born women ≥18 years were more than twice as likely to have never received a Pap test compared with U.S.-born women. Previous studies have also shown higher level of education predisposing women to more cervical screening as our study has found (16).

In terms of enabling factors, our study showed that women experiencing financial hardships (problems paying medical bills) were less likely to be *Non-Users* than adherent *Users*. This finding is inconsistent with a recent convenience sampling study of 970 women aged 21–65 which did not find a significant association between financial hardship (material, psychosocial, and behavioral aspects) and screening rates (31). Although we did not find inadequate use of screening to be significantly associated with financial hardship, adherence to cervical cancer screening may still be associated with problems paying bills. For preventive health services, such as cervical cancer screening, the Patient Protection and Affordable Care Act (ACA Section-2713) requires all ACA Marketplace and non-grandfathered private health plans to abolish patients cost-sharing (coinsurance or copayments) irrespective of whether the patient meets his/her deductible. However, previous research has underscored the fact that “full coverage” often encompasses only the initial test that screens for cancer, but the cancer screening process may require further testing to establish malignancy. Women may still be faced with financial barriers to completing the diagnostic process for cervical cancer following an abnormal initial test result (14). In that vein, our study still showed that indigent or uninsured women have greater likelihood of being *Non-Users* than *Users* and employed women are less likely to be *Inadequate-Users* than *Users*, independent of financial hardship. This corroborates previous studies on the associations between underscreening and over-screening and income, employment, and insurance coverage (17, 23, 31).

Our study revealed women with one or more children in the household vs. none are less likely to be *Non-Users*. According to the American Cancer Society, women who have had children (≥3) are at an increased risk of cervical cancer compared with those who have not, probably due to the increased exposure to HPV infection with sexual activity (32).

Usual source of care was a significant enabler of non-use or inadequate use. The literature shows inconsistencies regarding healthcare providers' adherence to USPSTF guidelines as over-screening is common practice among them (33–35).

The finding that well-visits in the past year (needs factor) are associated with lesser likelihood of non-use or inadequate use is consistent with the literature (36). Indeed, well-woman visits including annual check-up for gynecological and reproductive health focus on preventative care. Cervical screenings, Pap smears, and breast exams are three of the most common tests performed during well-woman visits.

Finally, our study unveiled an intriguing finding regarding HPV vaccination. While HPV vaccination was associated with lesser odds of non-use, it was associated with greater odds of inadequate use among eligible women. Studies have shown HPV vaccination is associated with decreased risk of cervical cancer (37) and have found women who receive HPV vaccination are more likely to participate in cervical cancer screening (depending on age of vaccination) (38, 39). It is plausible that women who have received the HPV vaccine may have a heightened sense of being protected against the virus (less at risk) and may be less prone to regimentally follow the recommended timeline for vaccinations. However, a study has shown HPV vaccinated Danish women perceived cervical cancer risk to be greater than unvaccinated women did, but the study found no associations between perceived cervical cancer risk and intention to participate in screening (40). The National Cancer Institute and the CDC encourage past HPV vaccinated populations to continue screening for cervical cancer because HPV vaccines do not protect against all HPV types that can cause cancer (41, 42). Vaccinated women are advised to follow the same cervical cancer screening guidelines as unvaccinated women. Future studies on screening recommendations in terms of harms and benefits for vaccinated women are warranted. Furthermore, studies on risk perceptions of cervical cancer screening among HPV vaccinated women stratified by factors associated with inadequate use (e.g., age group, race/ethnicity, uninsurance) are needed.

### 4.1 Study strengths and limitations

Findings from this study should be appraised in light of the following limitations: first, this study used cross-sectional data, and thus, causality cannot be established. Other limitations relate to recall bias, especially as the time window recommended by USPSTF becomes larger and as women age. We were limited with self-reported data, whereas medical records could have provided data less tainted by recall bias. Furthermore, the language around *Non-Users* and *Inadequate-Users* shifts the “blame” onto women, whereas healthcare providers might be the culprit for over or underscreening. The data used in our study did not allow us to clearly disentangle whether non-use or inadequate use was intentionally born by women or imposed by their providers. Despite these limitations, by using a conceptual framework and population data to explain factors associated with non-use and inadequate use separately, our study provides a more nuanced and complex explanation of cervical screening utilization among a representative sample of women. Future studies should explore mechanistic pathways (mediation and moderation) that lead to U.S. women's non-use and inadequate utilization of cervical cancer screening services.

## 5 Conclusion

Predisposing, enabling, and needs factors are differently associated with non-use and inadequate use of cervical cancer screening among eligible women aged 21–65 years. Understanding factors associated with the use, non-use, and inadequate use of cervical cancer screening is crucial to avoid or curb unnecessary tests, increased costs to both society and individuals, and the ill allocation of limited resources.

## Data availability statement

Publicly available datasets were analyzed in this study. This data can be found here: https://www.cdc.gov/nchs/nhis/2019nhis.htm. No datasets were generated for this study. Any analysis, interpretation, and/or conclusion based on the NHIS 2019 data is solely that of the authors. Opinions, conclusions, and recommendations expressed herein do not necessarily represent those of the National Center for Health Statistics or CDC, which are responsible for the data.

## Ethics statement

Ethical approval was not required for the study involving humans in accordance with the local legislation and institutional requirements. Written informed consent to participate in this study was not required from the participants or the participants' legal guardians/next of kin in accordance with the national legislation and the institutional requirements.

## Author contributions

M-RN: Methodology, Writing—original draft. PM: Writing—review & editing. EH: Writing—review & editing. NP-J: Writing—review & editing. HF: Conceptualization, Writing—review & editing.
